# LM14 defined medium enables continuous growth of *Trypanosoma cruzi*

**DOI:** 10.1186/s12866-014-0238-y

**Published:** 2014-09-10

**Authors:** Carla V De Paula Lima, Michel Batista, Fernanda G Kugeratski, Isabel M Vincent, Maurilio J Soares, Christian M Probst, Marco A Krieger, Fabricio K Marchini

**Affiliations:** Functional Genomics Laboratory, Carlos Chagas Institute-Fiocruz, Curitiba, Paraná Brazil; Cellular Biology Laboratory, Carlos Chagas Institute-Fiocruz, Curitiba, Paraná Brazil; The Wellcome Trust Centre for Molecular Parasitology, Institute of Infection, Immunity and Inflammation, College of Medical, Veterinary and Life Sciences, University of Glasgow, Glasgow, UK

**Keywords:** *Trypanosoma cruzi*, Chemically defined medium, Metacyclogenesis

## Abstract

**Background:**

*Trypanosoma cruzi*, the etiologic agent of Chagas disease, alternates between distinct morphological and functional forms during its life cycle. Axenic multiplication and differentiation processes of this protozoan parasite can be reproduced *in vitro*, enabling the isolation and study of the different evolutionary forms. Although there are several publications attempting the cultivation of *T. cruzi* under chemically defined conditions, in our experience none of the published media are capable of maintaining *T. cruzi* in continuous growth.

**Results:**

In this work we modified a known chemically defined medium for *Trypanosoma brucei* growth. The resulting LM14 and LM14B defined media enabled cultivation of five different strains of *T. cruzi* for more than forty passages until now. The parasite’s biological characteristics such as morphology and differentiation to metacyclic trypomastigotes were maintained when defined media is used.

**Conclusions:**

The establishment of a defined medium for *T. cruzi* cultivation is an important tool for basic biological research allowing several different approaches, providing new perspectives for further studies related to cell biology of this parasite.

**Electronic supplementary material:**

The online version of this article (doi:10.1186/s12866-014-0238-y) contains supplementary material, which is available to authorized users.

## Background

Chagas disease is a serious illness discovered by Carlos Chagas in 1909 [1], which is caused by the protozoan parasite *Trypanosoma cruzi*. About 7 to 8 million people are infected worldwide, mostly in Latin America where Chagas disease is endemic. In 2008, Chagas disease killed more than 10,000 individuals [2]. *T. cruzi* is usually transmitted to humans by the infected feces of blood-sucking triatomine bugs, although the parasites can also be transmitted through unconventional ways, including blood transfusions, organ transplants, congenital factors, laboratory accidents and oral transmission by ingestion of contaminated food [3].

*T. cruzi* undergoes remarkable morphological and physiological changes during development in both insect and mammalian hosts, adopting four distinct forms: epimastigotes and metacyclic trypomastigotes in the invertebrate vector and amastigotes and bloodstream trypomastigotes in vertebrate hosts [4]. Epimastigotes and amastigotes are replicative forms, whereas metacyclic and bloodstream trypomastigotes are infective, non-replicative forms.

Analysis of differentiation processes such as metacyclogenesis (differentiation from epimastigotes to metacyclic trypomastigotes) that lead to infectivity are of great interest. This differentiation can be reproduced *in vitro* [5–7], making it possible to isolate intermediate forms and study the time course of this process. Although *in vitro* metacyclogenesis is conducted under chemically defined conditions, the cultivation of epimastigote forms (pre-differentiation stage) is carried on in a complex high nutritive medium called LIT (Liver Infusion Tryptose) supplemented with 10% fetal bovine serum (FBS) [8,9]. Several components of this medium have an unknown composition, including yeast extract, liver infusion, tryptose and FBS. These components may contain growth factors, vitamins, hormones, proteins, lipids or other factors that can influence many biological aspects of the parasite. Furthermore, the quality and origin of the animal sources of these components are highly variable. These variations can potentially affect growth and differentiation rates, as well as responses to drugs, hindering the analysis of experimental results conducted in this context.

Use of a chemically defined medium for the cultivation of *T. cruzi* could improve the current scenario helping to develop more uniform and standardized assays. For example, in a drug discovery context, it would bring more reproducibility and reliability to the testing of new trypanocidal drugs, as well as studying their effects and their mechanisms of action in this parasite [10–12]. In addition, use of a defined medium is essential for many useful assays, such as perturbation of medium components, cellular metabolic labeling and measurement of metabolites secreted by cells in the medium.

Several articles have already been published showing the cultivation of different strains of *T. cruzi* epimastigotes in defined media. In 1977, Azevedo and Roitman published a research note [13] communicating the continuous cultivation of Y strain in a defined medium – AR-103 – which was developed based on a previous defined medium for cultivation of *T. brucei* procyclic cells named HX25 [14]. In 1975, Anderson and Krassner [15], as well as Cross and coworkers [16], showed successful cultivation of Costa Rica and Sonya strains, respectively, in HX25 defined medium. Avila and coworkers cultivated strains Y, Ma, Fl and Marin-1 of *T. cruzi* in a defined medium containing only D-glucose, inorganic salts, some vitamins, nucleotides and bovine liver catalase [17]. Later they developed a minimal medium based on the previous, where nucleotides and vitamins had been excluded [18]. However, these results were criticized by O’Daly and Rodriguez [19], since they showed the existence of 25 to 30 protein bands as well as DNA and RNA polymers contaminating the solution of bovine liver catalase used in the medium.

Thereafter, few published reports showed the cultivation of *T. cruzi* in a defined medium, and all of them only for a single passage, highlighting the challenge of continuously cultivating this parasite over long periods under defined conditions.

In the present work we demonstrate a continuous cultivation of *T. cruzi* epimastigote cells in two defined media derived from HX25M [20], named LM14 and LM14B. With these media, we cultivate five different strains of *T. cruzi* for more than forty passages. Morphology in defined conditions was maintained, as well as the parasite’s ability to differentiate to infective forms. Defined media described herein are powerful tools, indispensable for the application of several methods that could not be performed using non-defined media.

## Results and discussion

### HX25M and AR-103 media are not sufficient for *T. cruzi* cultivation

LITB+FBS is a complex high nutritive medium in which *T. cruzi* is routinely cultivated. The high complexity of the medium makes it infeasible to modify, removing or replacing its components. For this purpose, we tested the growth and maintenance of *T. cruzi* cells in both defined media AR-103 and HX25M. HX25M was developed to cultivate *T. brucei* cells [20], and has also been used to cultivate *T. cruzi* [15,16], while AR-103 was developed based on HX25M and is used only for *T. cruzi* cultivation [13].

**Figure 1:**
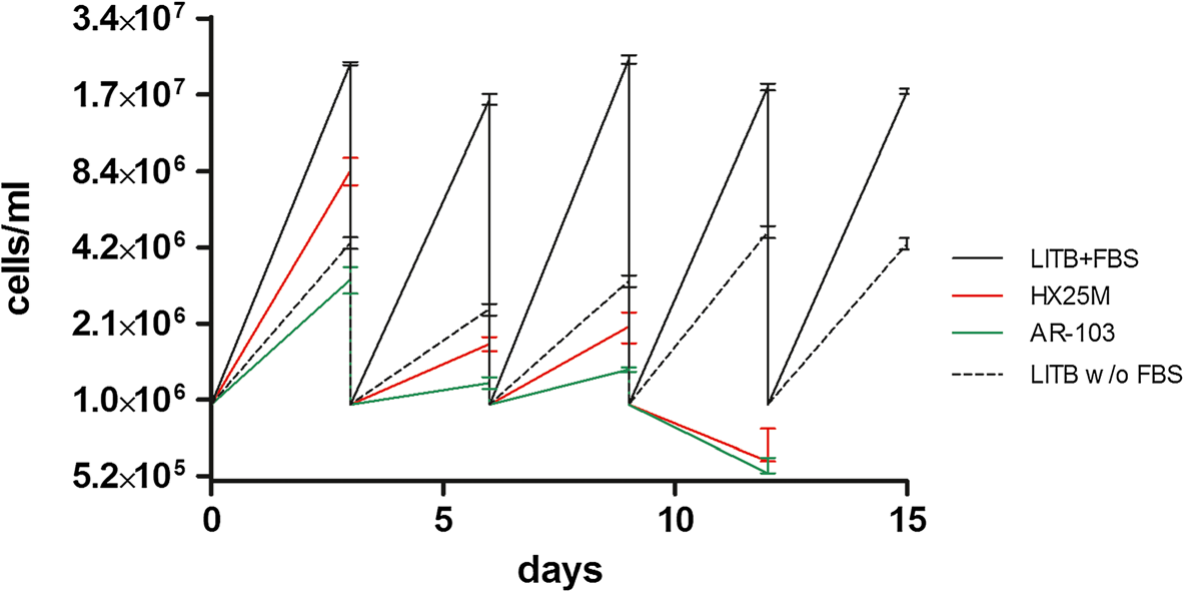
**Growth comparison between defined (HX25M and AR-103) and complex (LITB+FBS and LITB without FBS) media.** Epimastigote cells counts were performed every three days, then all cultures were diluted to 1e + 06 cells/ml (start point of cultivation). Values plotted refer to the average of three biological replicates counted every cell passage after three days of cultivation. Cell cultivation in LITB+FBS (black line) and LITB without FBS (dashed line) was maintained for five passages of three days, whereas defined media HX25M (red line) and AR-103 (green line) did not support cell growth after the third passage.

To evaluate the growth rate of cells cultivated in defined media, AR-103 and HX25M media were tested for *T. cruzi* cultivation (strain *Dm*28c). Growth rates after three days of cultivation were compared with LITB+FBS medium, the routinely used medium for *T. cruzi* cultivation, and LITB without FBS, once FBS is absent in defined media. Cultures were maintained over five passages (Figure 1), all starting from 1e + 06 cells/ml. After the fourth passage, there was no cell growth in both HX25M or AR-103, indicating that these media do not support *T. cruziDm*28c cell growth in these conditions. We obtained similar results in independent experiments performed using different batches of media at different times and using different types of flasks and plates (data not shown).

### Putrescine allows continuous growth of *T. cruzi* in defined media

*T. cruzi* epimastigotes are auxotrophic for diamines, such as putrescine since *T. cruzi*, unlike other trypanosomatids, lacks the genes encoding either ornithine decarboxylase or arginine decarboxylase (ODC and ADC), enzymes responsible for putrescine biosynthesis [21–23]. Diamines are essential for cell proliferation, differentiation and macromolecular synthesis [24]. *T. cruzi* is able to convert exogenous putrescine to trypanothione and cadaverine to homotrypanothione, antioxidants which are unique to the kinetoplastids and fulfill many of the roles ascribed to glutathione in other eukaryotic cells [25].

**Figure 2:**
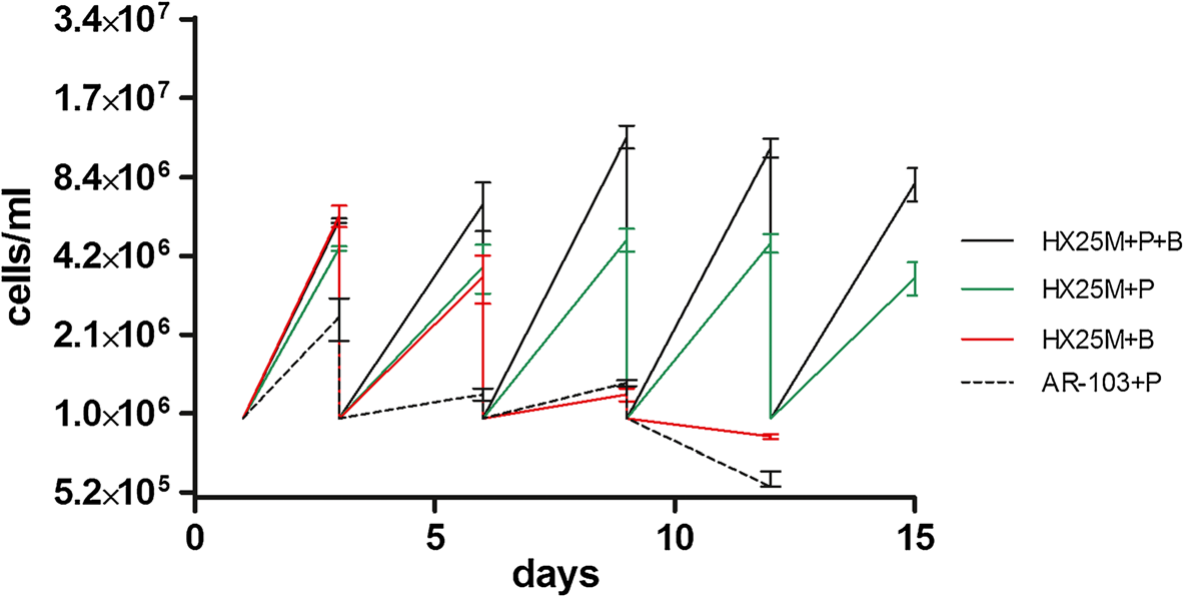
**Putrescine and biopterine test in cell culture.** Putrescine was added at 10 μM final concentration in defined media HX25M (HX25M+P) and AR-103 (AR-103+P). Biopterin was added at final concentration of 1 μM in defined medium HX25M with (HX25M+P+B) or without (HX25M+B) putrescine. Epimastigote cells counts were performed every three days, then all cultures were diluted to 1e + 06 cells/ml (start point of cultivation). Values plotted refer to the average of three biological replicates counted every cell passage after three days of culture. Epimastigote cultures were maintained for six passages in HX25M+P defined medium (green line), whereas cells cultivated in AR-103+P defined medium (dashed line), even with putrescine, died after the third passage. Epimastigotes cultivated in HX25M+B medium (red line) died after the third passage. Epimastigotes cultivated in HX25M+P+B medium (black line) showed a significant increase in growth rate compared to HX25M+P medium (*P* < 0.01) (green line).

AR-103 and HX25M media lack diamines in their formulations, requiring the addition of at least one of them. Therefore, we tested cultivation of *T. cruzi* in defined media supplemented with 10 micromolar of putrescine (HX25M+P and AR-103+P). Figure 2 shows that parasites cultivated in AR-103+P died after the fourth passage (dashed line), whereas parasites in HX25M+P exhibited continuous growth, with increasing growth rate after several passages (green line).

These results show that defined media AR-103 and HX25M were not sufficient for continuous cultivation of *T. cruzi* cells, contradicting the results obtained by Azevedo and Roitman [13], Anderson and Krassner [15] and Cross et al. [16], being putrescine an essential compound for this purpose. AR-103 medium, even with putrescine, does not sustain continuous growth of *T. cruzi* in our hands.

Putrescine is greater than 97% pure and since addition of this compound alone, to a final concentration of 10 micromolar, was sufficient to convert medium from non-growth sustaining to growth sustaining we can rule out that any contaminant as being responsible for the acquired growth.

### Biopterin accelerates *T. cruzi* growth in defined medium

Biopterin is a biologically significant pterin that functions as an essential cofactor for several enzymes involved in processes including hydroxylations, ether-lipid cleavage, and nitric oxide synthase [26–28]. Some trypanosomatids, like *Leishmania*, *Crithidia* and *T. brucei*, are unable to synthesize the pterin moiety from GTP (guanosine triphosphate) and thus must acquire pteridines from the host by salvage mechanisms [29–31].

Biopterin was added to defined medium, with (HX25M+P+B) or without (HX25M+B) putrescine, at a final concentration of 1 μM. The growth rate was measured by counting at every three days of cultivation for five passages. Biopterin was not sufficient for continuous cultivation of *T. cruzi* without putrescine (Figure 2, HX25M+B, red line), but addition of biopterin to HX25M+P medium significantly increased the growth rate of the parasites compared to medium without biopterin HX25M+P (*P* < 0.01) (Figure 2, HX25M+P+B, black line).

The results presented above show that putrescine is required for *T. cruzi* epimastigote cultivation and biopterin shows growth stimulation of these parasites. These modified media were named LM14 (HX25M+P) and LM14B (HX25M+P+B).

**Figure 3:**
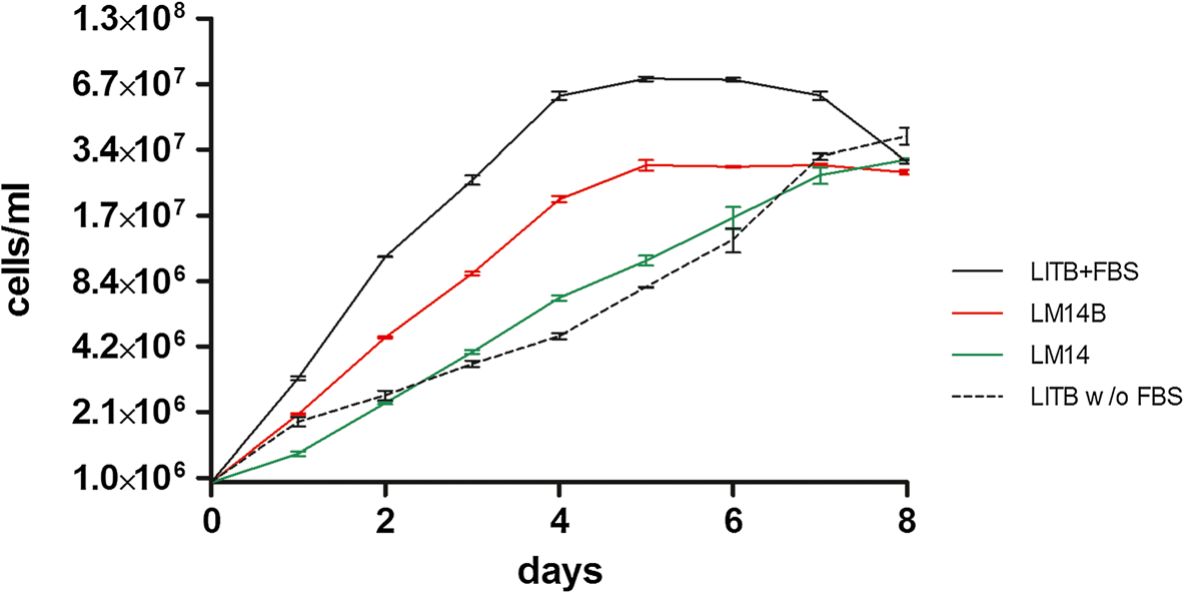
***In vitro*** **growth curve of** ***T. cruzi*** **in defined (LM14 and LM14B) and complex (LITB+FBS and LITB without FBS) media.** Epimastigote cells counts were performed at every 24 hours, for eight days (beginning of cell death). Values plotted refer to the average of three biological replicates. Epimastigotes cultivated in defined medium LM14B (red line) had a similar profile of growth curve to parasites cultivated in LITB+FBS (black line), reaching the stationary phase at 5^th^ day, as well as a similar growth curve was observed for epimastigotes cultivated in LM14 (green line) and LITB w/o FBS (dashed line).

Comparisons between parasite growth curves in defined and complex media were obtained by cultivating epimastigote forms at an initial density of 1e + 06 cells/ml, until they reached stationary phase or cell death. Stationary growth phase was reached on the 7^th^ day for LM14 (2.8e + 07 cells/ml) and LITB without FBS (3.4e + 07 cells/ml) (Figure 3). As observed in LITB+FBS cultures, stationary growth phase of parasites cultivated in LM14B is reached at the 5^th^ day (3e + 07 cells/ml) (Figure 3). Although growth rate of parasites cultivated in LM14B is slower than in LITB+FBS, when compared to cells growing in LITB without FBS it is notably faster. This result suggests that LM14B defined medium is better suited for *T. cruzi* growth than the standard medium LITB when FBS is absent.

To verify whether there are any undefined protein and/or DNA fragment present, we performed a SDS-polyacrylamide electrophoresis as well as an agarose gel electrophoresis of tested medium (data not shown). Only a 60–70 kDa protein was observed, mass related to albumin [32], which is added to the defined medium (bovine serum albumin – BSA). Also we did not observe any DNA fragment in the defined media. These results indicate that there are no protein or DNA contaminants in the tested defined medium, at least with the sensibility reached by the detection methodology used.

### Parasites cultivated in LM14 and LM14B defined media can differentiate *in vitro* into metacyclic trypomastigotes

**Figure 4:**
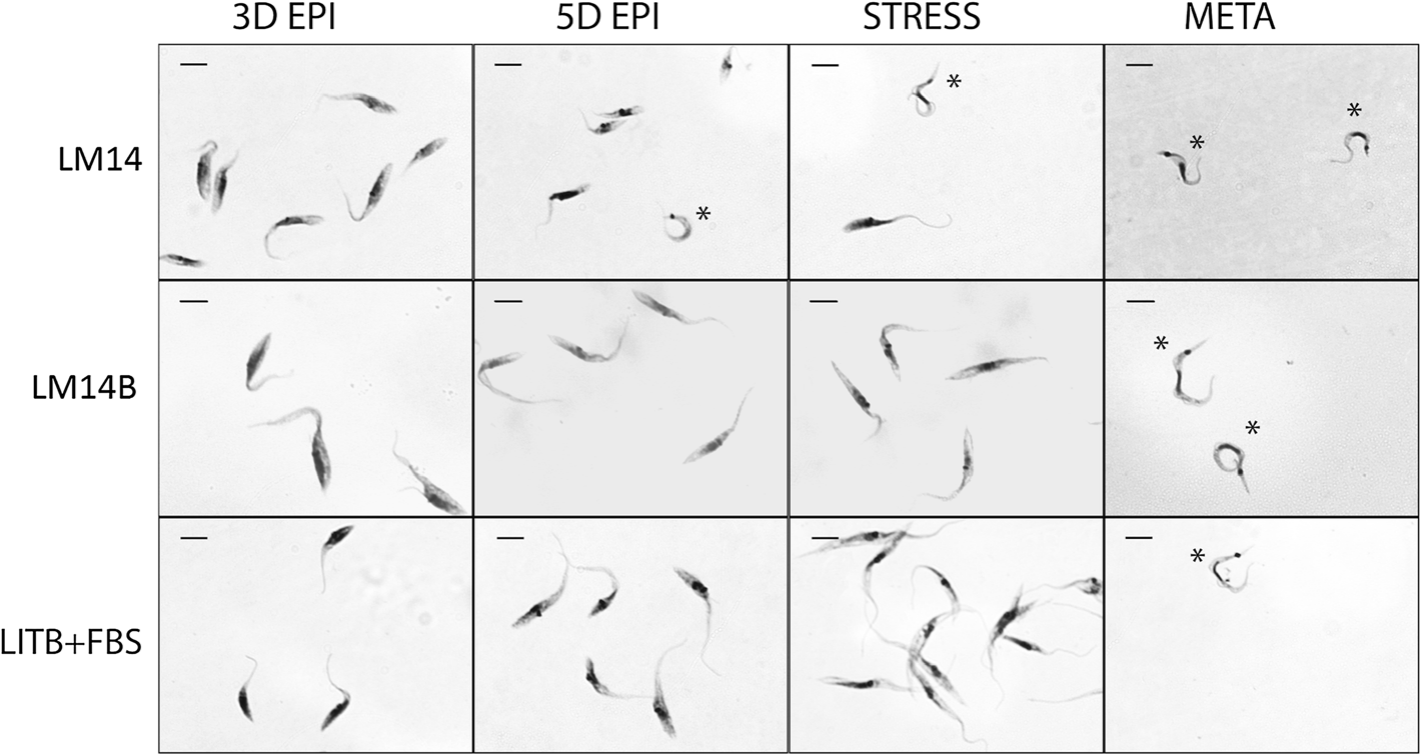
**Parasite morphology during metacyclogenesis in defined (LM14, LM14B) and complex (LITB + FBS) media.** Cells were panoptic stained (Laborclin, Pinhais, Parana, BR). Images are related to epimastigotes after three days of cultivation (3D EPI), five days of cultivation (5D EPI), parasites in TAU medium for nutritional stress (STRESS) and metacyclic trypomastigotes after *in vitro* metacyclogenesis (META). The upper panel shows parasites from defined medium LM14, where spontaneous metacyclic trypomastigotes are observed in the early stationary growth (5D EPI and STRESS). The middle panel shows parasites from the defined medium LM14B in different stages of differentiation. The lower panel shows parasites from LITB+FBS medium (complex medium). Metacyclic trypomastigotes are indicated with an asterisk. Size bar indicates 10 μm.

*In vitro* metacyclogenesis was performed to test the biological capability of *T. cruzi* cells to differentiate after cultivation in the defined medium. Epimastigotes forms cultivated in LM14 defined medium showed a differentiation rate of about 30% to metacyclic trypomastigotes after 72 hours of differentiation, a similar differentiation rate found in parasites from LITB+FBS medium (about 32% of metacyclic trypomastigotes) under the same conditions. We also observed spontaneous metacyclic trypomastigote forms from the third day of culture in LM14 medium, before the stimuli for differentiation (Figure 4 upper panel, 5D EPI and STRESS). Interestingly, parasites cultivated in LM14B defined medium, although exhibiting a better growth rate than in LM14 and absence of spontaneous metacyclic trypomastigotes before the stress stimuli, they showed a much lower differentiation rate, about 18% of metacyclic trypomastigotes after 72 hours of differentiation. This event can be compared to that observed in *Leishmania major*, where a tetrahydrobiopterin (the active form of biopterin) deficiency in promastigote forms increased differentiation into the mammalian-infectious metacyclic promastigote form, probably indicating a role of pterins in resistance to oxidative stress, but the underlying mechanism remains obscure [33,34].

Although parasites from LM14B exhibit a lower differentiation rate compared to those in LITB+FBS, the process is still occurring in this condition and can be performed with parasites from LM14 medium with a higher differentiation rate. This represent a strong indication that the biological capabilities of *T. cruzi* in our defined medium are maintained.

We also analyzed the ultrastructure of *T. cruzi* epimastigotes cultivated in LITB+FBS, LITB without serum and LM14B media to verify possible morphological alterations in cells cultivated in defined medium, when compared with conventional complex medium. Analysis of *T. cruzi* ultrathin sections by transmission electron microscopy showed no visible alteration in cell shape or intracellular organelle content (data available in Additional file 1), showing that the morphology of *T. cruzi* cultivated in LM14B is maintained.

### Different *T. cruzi* strains can be cultivated in LM14B defined medium

*T. cruzi* is a highly polymorphic specie. The biological, biochemical and genetic diversity of *T. cruzi* isolates has long been recognized [35–37]. Over the years, several approaches have been used to separate the *T. cruzi* population into different groups, or discrete typing units (DTUs). Recently, Zingales and coworkers reorganized by consensus the nomenclature of these DTUs as TcI-TcVI [38].

**Figure 5:**
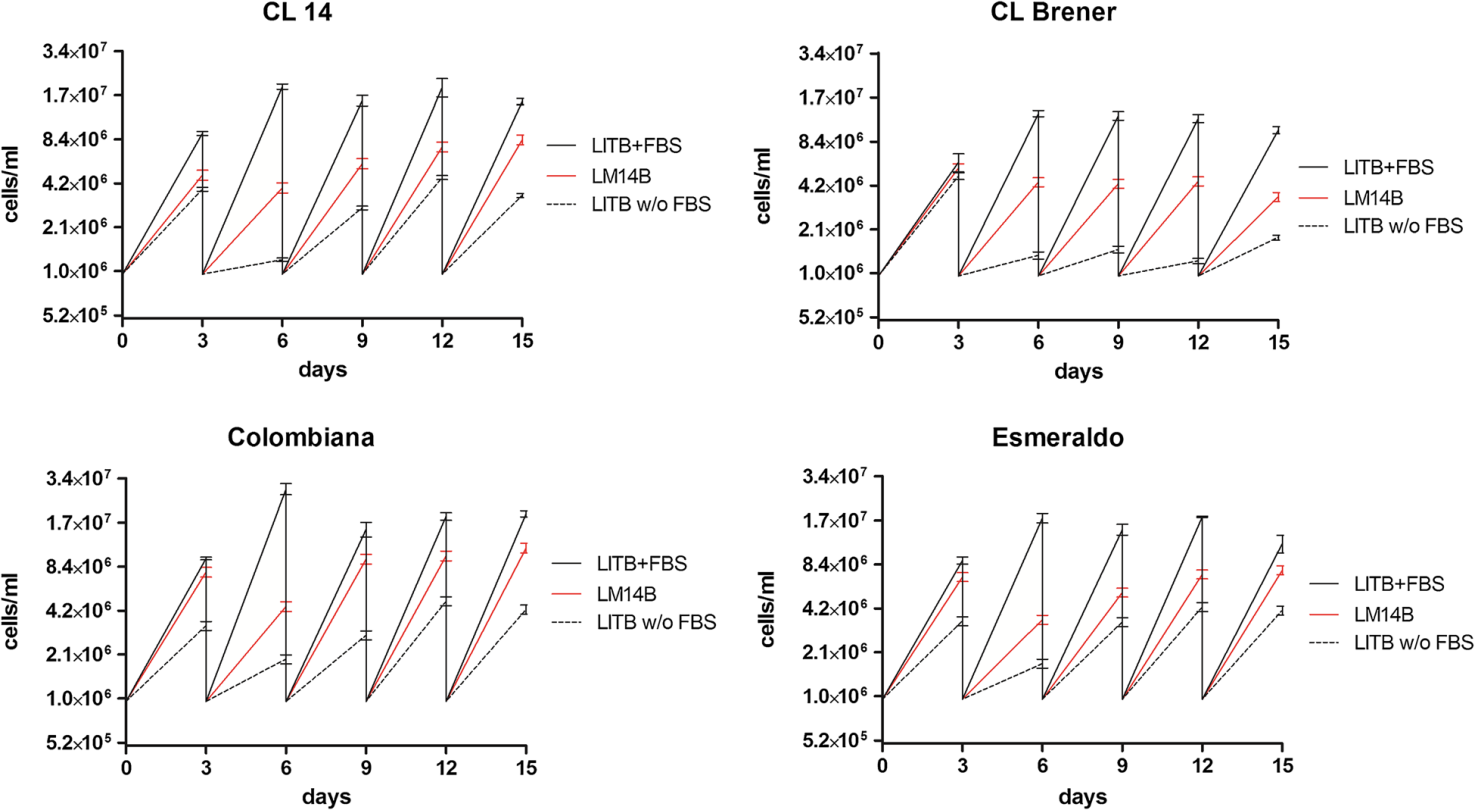
**Growth evaluation of different** ***T. cruzi*** **strains in LM14B defined medium.** Epimastigote counts were performed at every three days, then all cultures were diluted to 1e + 06 cells/ml (start point of cultivation). Values plotted refer to the average of three biological replicates, with counts at every third day of culture.

We selected strains from different DTUs to test their suitability to grow in LM14B defined medium. They were: Colombiana and *Dm*28c (DTU I), Esmeraldo (DTU II), CL14 and CL Brener (DTU VI). Our findings clearly demonstrate that LM14B medium was efficient for cultivating all tested *T. cruzi* strains in defined conditions, even better than cultivation in LITB medium without FBS (Figure 5).

## Conclusion

Our data report the development of a defined medium for *T. cruzi* cultivation. Despite previously published work reporting sustained cultivation of *T. cruzi* in defined medium, we could not reproduce such results. Therefore, to our knowledge, LM14 and LM14B are the only chemically defined medium capable of continuous axenic *T. cruzi* cultivation (more than forty passages until now for all the tested strains) while maintaining *T. cruzi* morphology and ability to differentiate. Both defined media described herein present important advances to study the cell biology of this parasite, since they enable metabolic labeling, drugs research and evaluation, metabolic studies, among other powerful methods, providing new perspectives for further studies related to *T. cruzi* biochemistry.

## Methods

### Defined media preparation

All components of both AR-103 and HX25M defined media were individually dissolved in appropriate solvent, at concentrations under each limit of solubility. The required amount of each component for 1 liter of medium was added to 500 ml of distilled water and mixed under constant agitation at room temperature until complete dissolution. After, the medium pH was adjusted to 7.2 and the volume was completed to 900 ml with water. The volume was set to 1 liter with water. Finally, the medium was sterilized using a 0.22 μm filter.

The final LM14B medium formulation and method of preparation can be found in Additional file 2.

#### *Trypanosoma cruzi* growth

The experiments were performed using the *T. cruzi* strain *Dm*28c [7]. Culture epimastigote forms were maintained at 28°C in LITB medium supplemented or not with 10% fetal bovine serum (FBS) [8,9], by passages every three days from an initial density of 1e + 06 cells/ml in fresh medium.

Cell cultures in defined media were obtained from cultures previously cultivated in LITB+FBS medium. In order to eliminate LITB+FBS medium before cultivation in defined media, cells were washed twice in sterile phosphate saline buffer (137 mM NaCl, 2.7 mM KCl, 4.3 mM Na_2_HPO_4_, 1.5 mM KH_2_PO_4_). Media evaluation was performed with three biological replicates and passages every three days of 1e + 06 cells/ml. After confirmation of continuous and stable growth of cultures over several passages, a growth curve was obtained with three biological replicates, using an initial concentration of 1e + 06 cells/ml, and daily counting until cultures reached stationary phase, or until start of cell death.

All cultures were maintained in 15 ml conical centrifuge tubes, at a final volume of 3 ml. Epimastigote cells counts were performed using an automatic counter (Z2 Coulter® – Beckman Coulter).

To test the suitability of LM14B to maintain growth of different strains of *T. cruzi,* the CL14, CL Brener, Colombiana and Esmeraldo strains were tested [39]. All were cultivated in LITB+FBS, LITB without FBS and LM14B medium with passages every three day from an initial density of 1e + 06 cells/ml in fresh medium. Cells were harvested from LITB+FBS media by centrifugation at 7,000 × g for 5 min at 20°C and washed in sterile phosphate saline buffer before starting the experiment.

Data were compared via two-way analysis of variance (ANOVA) followed by Bonferroni multiple range test for statistically significant differences at p < 0.05.

#### *In vitro* metacyclogenesis

Aiming to verify if biological characteristics of parasites are maintained in defined medium, we performed *in vitro* differentiation from epimastigotes forms (replicative) to metacyclic trypomastigotes forms (infective), a process known as metacyclogenesis [5]. For this purpose, epimastigotes were harvested from LITB+FBS, LM14 and LM14B media after 5 days of culture (early stationary phase), by centrifugation at 7,000 × g for 5 min at 20°C. Epimastigotes were then submitted to nutritional stress by incubation at 28°C in TAU medium (190 mM NaCl, 17 mM KCl, 2 mM MgCl_2_, 2 mM CaCl_2_, 8 mM phosphate buffer pH 6.0) for 2 hours at a concentration of 5e + 08 cells/mL. Next, cultures were diluted 1:100 in TAU3AAG medium (TAU supplemented with 10 mM L-proline, 50 mM L-sodium glutamate, 2 mM L-sodium aspartate, and 10 mM D-glucose). After 96 h of incubation, differential counts between epimastigotes and metacyclic trypomastigotes forms were performed in Neubauer chamber to determine the differentiation rates.

## Additional files

Additional file 1:
**Ultrastructural morphology of** ***T. cruzi*** **epimastigotes under cultivation in different culture media.**
Additional file 2:
**LM14B preparation method.**

## Abbreviations

FBS: Fetal bovine serum
HX25M+P: HX25M medium supplemented with putrescine (named as LM14 in this article)
HX25M+B: HX25M medium supplemented with biopterine
HX25M+P+B: HX25M medium supplemented with putrescine and biopterine (named as LM14B in this article)
DTU: Discrete type units
