# Supplementary material for: LM14 defined medium enables continuous growth of Trypanosoma cruzi
Source: BMC Microbiol. 2014 Sep 10;14:238. doi: 10.1186/s12866-014-0238-y (PMC4172853; doi:10.1186/s12866-014-0238-y)

**Supporting Information**

**Figure S1. Ultrastructural morphology of *T. cruzi* epimastigotes under cultivation in different culture media.** *T. cruzi* epimastigotes were cultivated in LITB+FBS medium (upper panel), LITB without FBS (middle panel) or LM14 defined medium (lower panel) and then processed for routine transmission electron microscopy. No remarkable morphological alterations were observed in cell shape and cell organelles such as flagellum (F), kinetoplast (K), nucleus (N), mitochondrion (M) and reservosomes (R), when parasites cultivated in LITB+FBS and LM14 media were compared. Parasites cultivated in LITB medium without serum presented less dense reservosomes.

**LITB+FBS**


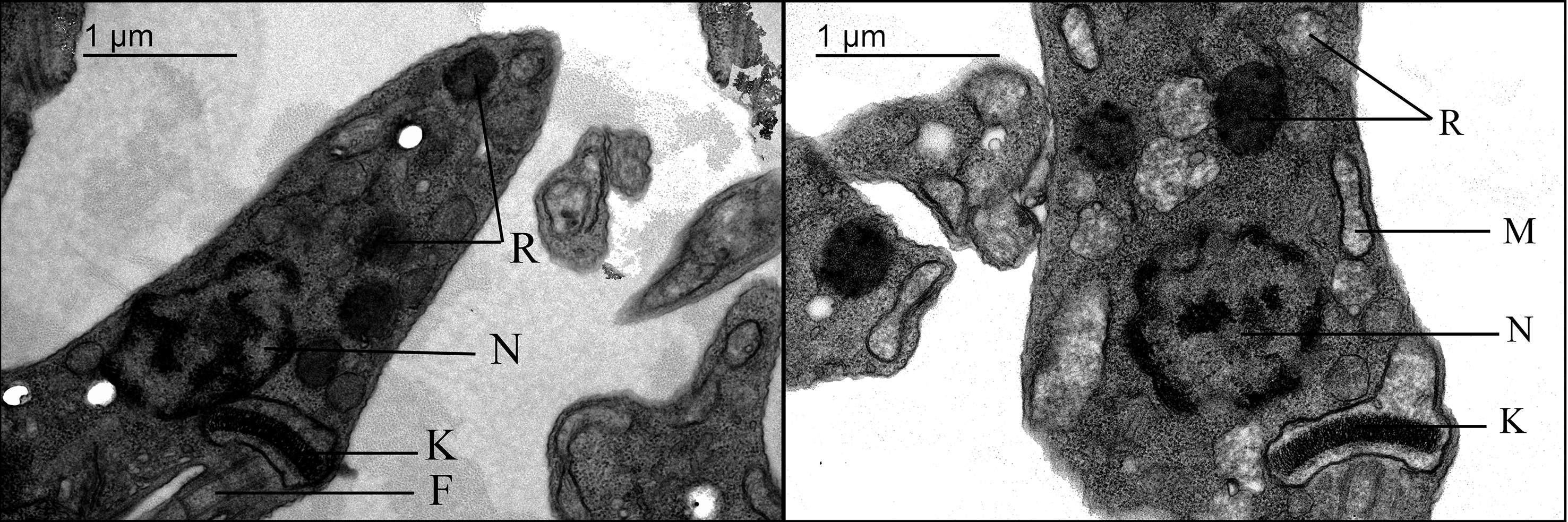


**LITB without FBS**


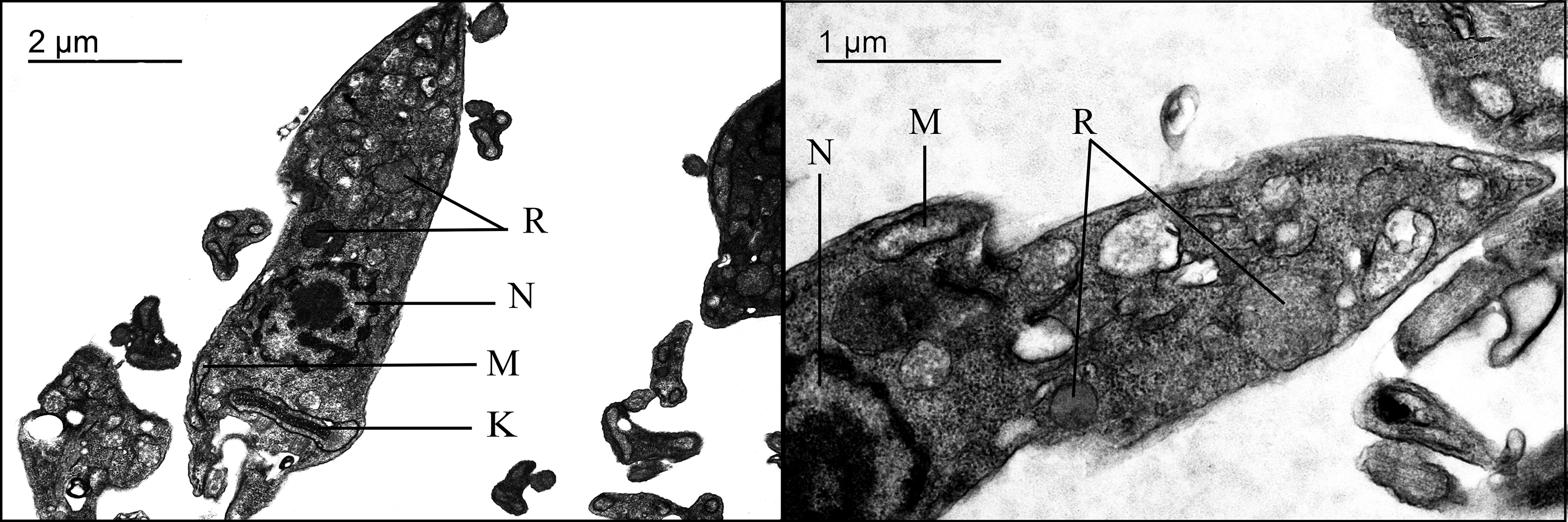


**LM14**


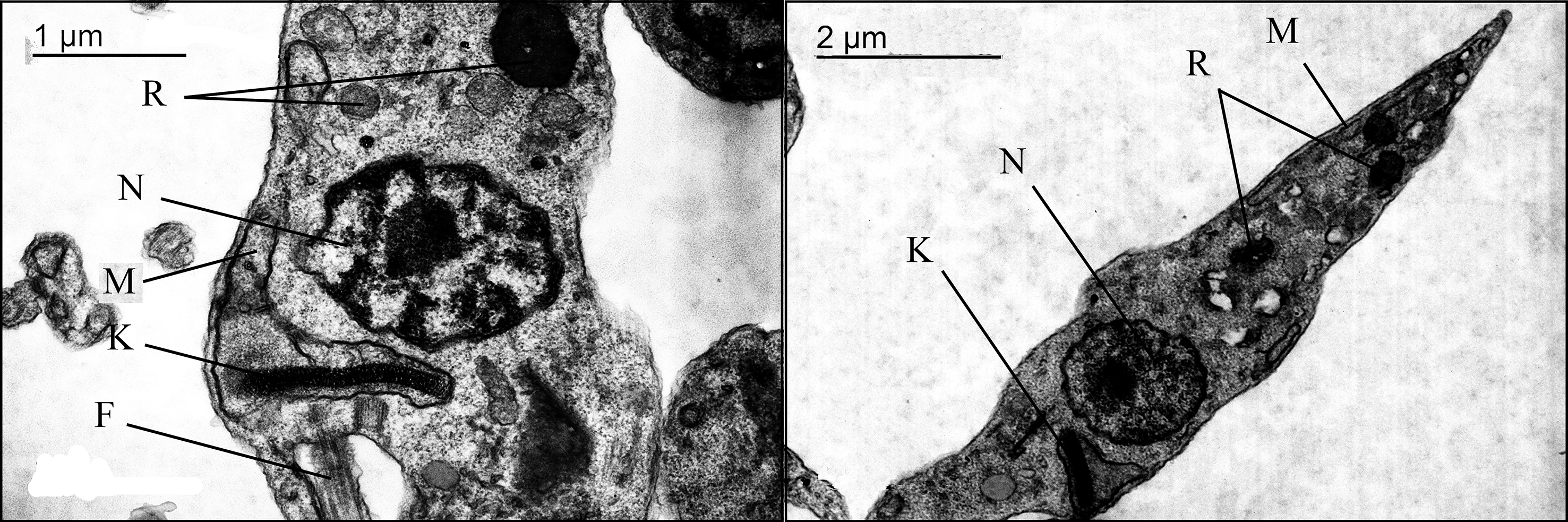

Supplement: Additional file 1: — Ultrastructural morphology of T. cruzi epimastigotes under cultivation in different culture media. [file 12866_2014_238_MOESM1_ESM.doc]
